# Explaining rule of rescue obligations in healthcare allocation: allowing the patient to tell the right kind of story about their life

**DOI:** 10.1007/s11019-021-10047-y

**Published:** 2021-09-12

**Authors:** Sean Sinclair

**Affiliations:** University of Leeds, IDEA Centre, Leeds, UK

**Keywords:** Rule of rescue, End of life premium, Narrative, Identifiability, Life stories, Death

## Abstract

I consider various principles which might explain our intuitive obligation to rescue people from imminent death at great cost, even when the same resources could produce more benefit elsewhere. Our obligation to rescue is commonly explained in terms of the identifiability of the rescuee, but I reject this account. Instead, I offer two considerations which may come into play. Firstly, I explain the seeming importance of identifiability in terms of an intuitive obligation to prioritise life-extending interventions for people who face a high risk of an early death, and I explain this in turn with a fair innings-style principle which prioritises life-extending interventions for people expected to die young. However, this account is incomplete. It does not explain why we would devote the same resources to rescuing miners stuck down a mine even if they are elderly. We are averse to letting people die suddenly, or separated from friends and family. And so, secondly, I give a new account that explains this in terms of narrative considerations. We value life stories that follow certain patterns, classic patterns which are reflected in many popular myths and stories. We are particularly averse to depriving people of the opportunity to follow some such pattern as they approach death. This means allowing them to sort out their affairs, say goodbyes to family and friends, review their life, or come to terms with death itself. Such activities carry a lot of meaning as ways of closing our life story in the right way. So, for someone who has not been given much notice of their death, an extra month is worth much more than for other patients. Finally, I review the UK National Health Service's end of life premium, which gives priority to patients with short life expectancy. I suggest it falls short in terms of such considerations. For example, the NHS defines its timings in terms of how long the patient can expect to live as at the time of the treatment decision, whereas the timings should be specified in terms of time from diagnosis.

## Introduction

In this paper I will consider whether a "rule of rescue" obligation is ever applicable in healthcare allocation. By this I mean (roughly) an obligation to help an individual whose life is imminently at risk, where the intervention is relatively costly and therefore does not maximise the expected benefit we can produce with the resources at our disposal. Outside healthcare, such an obligation sometimes seems applicable. For example, consider the Chilean government's 2010 rescue of the miners stuck down the Copiapó mine,^1^ or the Australian government's 1997 rescue of the lone yachtsman Tony Bullimore,^2^ lost in the Southern Ocean after his boat had capsized. Suppose that on one of these occasions, a minister had announced that she would not authorise the rescue because the cost per life year was estimated to be 20% above the normal threshold for healthcare interventions, and she intended to use the budget to extend more lives via healthcare interventions. This would have prompted strong protests from the public, indicating that intuitively at least, a strong obligation was applicable despite the relatively poor cost-effectiveness of the rescues.

## Evidence that some healthcare debates are driven by rule of rescue intuitions

I begin my investigation by considering some cases where healthcare policy debates seem to have been driven by rule of rescue intuitions. The first case involves the occasions when healthcare policy-makers refuse funding for a new, life-extending cancer drug on grounds of expense. The emotive response is reminiscent of the kind of response one might expect to the imaginary government minister in the Chilean miners case or the Tony Bullimore case. For example, in the UK, prior to NICE's introduction of its end of life premium in 2009 (prioritising patients with short life expectancy), NICE regularly came up against public opposition to its strict application of benefit maximising criteria. In 2005, after NICE refused funding for the breast cancer drug Herceptin, Health Secretary Patricia Hewitt questioned a ruling by Stoke local health bosses not to fund the drug for a patient. After mounting pressure they reversed the decision.^3^ Other local health bosses also fell into line with the Stoke decision, and started funding Herceptin. I take this was largely in response to a sense that the objectors had public opinion behind them. I also construe public opinion as an intuition that the refusal of funding was wrong. Cases like this motivated the introduction of NICE's end of life premium not long after, in 2009, whereby NICE relaxed the cost-effectiveness threshold for life-extending treatments for patients expected to live less than two years without treatment.

A well-known case from the US illustrates similar intuitions at work. This is the case of the first Oregon healthcare plan of the early 1990s. According to a widely-cited analysis by Hadorn, the plan foundered because it was guided entirely by cost–benefit comparisons:

Specific examples taken from a single page of the 161-page list illustrate the problem. Surgical treatment for ectopic pregnancy and for appendicitis are rated just below, or as less important than, dental caps for "pulp or near pulp exposure" and splints for temporomandibular joint disorder, respectively. This priority order occurred despite the fact that the former surgical procedures are virtually 100% effective in treating otherwise generally fatal conditions, while the latter conditions are minor and may resolve even without treatment. This counterintuitive preference order did not occur as a result of faulty data, as was suggested by OHSC, or by chance, but as an inevitable consequence of the application of cost-effectiveness analysis.^4^

As noted, in Hadorn's view the problem was not faulty data, nor faulty analysis. Hadorn judges that the estimates of cost-effectiveness for various treatments appear reasonable, but:

These reasonable estimates did not translate into reasonable (relative) priority ratings, however. Although both surgical procedures for appendectomy and ectopic pregnancy were correctly estimated to entail a far higher level and duration of benefit than either of the two minor treatments, the relatively high costs of surgery effectively neutralized these outcome considerations, producing nearly identical priority ratings for all four treatments.^5^

But although Hadorn thinks the ranking of treatments was based on a reasonable analysis of their cost-effectiveness, still, he argues that the attempt to apply cost-effectiveness analysis rigorously foundered in the face of a very human propensity:

people cannot stand idly by when an identified person's life is visibly threatened if effective rescue measures are available.^6^

Once again, diseases which threaten imminent death seem to have a special status in terms of ordinary intuitions, and these intuitions are strong. Policy-makers ignore them at their peril.

There are also various other cases in which a similar principle seems to be at work. For example, consider the privileged status of treatment vis-à-vis prevention. One estimate was that for a given healthcare budget, you could get 10 quality-adjusted life years (QALYs) from dialysis of kidney patients or 1197 QALYs from stop-smoking campaigns.^7^ Yet everyone who needs dialysis gets it, when we could spend the money on increasing the reach of public health campaigns that would save more lives. This is difficult to explain in terms of non-rescue considerations, given that the health outcomes prevented by the respective interventions are comparably severe.^8^ Once again, it seems that patients at risk of imminent death merit special status, intuitively.

## Can we explain rule of rescue obligations in terms of the identifiability of the prospective victim?

I now propose to investigate whether we can vindicate rule of rescue intuitions. The most popular candidate for a morally relevant feature in rule of rescue cases is the identifiability of the prospective beneficiaries of the rescue.^9^ I will consider this account only briefly before rejecting it. However, I must acknowledge that many descriptive papers characterising the human disposition to perform a rescue in paradigm rescue cases take identifiability to be the key feature of the initial conditions in which rescue behaviour is triggered. For example, Hadorn's characterisation of the behaviour in question is that "people cannot stand idly by when an identified person's life is visibly threatened."^10^ And focusing on normative accounts, writers who defend moral relevance of identifiability include McKie & Richardson,^11^ Orr & Wolff,^12^ Largent & Pearson,^13^ and Schöne-Seifert.^14^ Mark Sheehan also offers a qualified defence.^15^

But identifiability does not seem to be a morally relevant feature. A number of writers have objected against it. For example, McKie and Richardson ask "Why should those who are lucky enough or manipulative enough to attract media attention be thought to have a special claim on resources?"^16^ The point is that intuitively, those who get media coverage do not have *any* more right to tax-funded healthcare resources than those who don't, all else being equal.

Closer to home, in the domain of in healthcare allocation, consider a case from the UK where patients are sometimes identifiable to policy-makers: the case of panels that review Individual Funding Requests. IFRs are requests submitted by hospital consultants to Clinical Commissioning Groups on behalf of patients who have an exceptional characteristic such as a comorbidity, so they do not fit on one of the standard care pathways, but the consultant thinks a treatment will be beneficial for them. For IFR panels, patients are identified. In some cases, patients are allowed to attend the panels. In other cases, they are identified by name. Even if they are not, they are at least picked out individually to the same extent as, say, miners stuck down a mine might be for a politician deciding whether to launch an operation to get them out, in that they constitute known individuals subject to a known risk. Suppose that one or other of these forms of identification is available to panel. Still, intuitively it would seem quite reasonable for the panel to apply the same cost-effectiveness criteria as are applied in standard commissioning decisions applying to unidentified patients. To the extent that data is available,^17^ it would not seem impermissible to consider what it implies for the standard cost-effectiveness criteria, especially if the panel are allocating money from the same overall budget as in standard commissioning decisions. In fact, it would seem quite unfair to other patients if IFR decision-makers *didn’t* apply the same criteria as are applied to other patients, to the extent that this is possible.^18^

To make this point vivid, suppose that policy-makers approve an IFR for a given type of case, and then decide they need to formulate a standard care pathway for that type of case. They formulate criteria based on the same broad cost-effectiveness criteria as applied in all other therapy areas. So, when the new pathway is published the week after the IFR case, a hospital consultant tells a newly-diagnosed, clinically identical patient that according to the newly published criteria, the patient will not get funding for the drug they need. The patient knows that the first patient was clinically identical, and was funded, and therefore asks why he was refused. When the hospital consultant seeks clarification from policy-makers, they reply that they met the first patient but not the second one, and so different criteria were applied. This would seem very unfair. So, even when patients are identifiable to policy-makers, it does not seem that policy-makers are subject to any rule of rescue obligations which might be said to be triggered by such identifiability.

So, prima facie, identifiability does not look like it can vindicate our intuitions in rule of rescue cases.

One line of response is to say that our rule of rescue behaviour is not justifiable; it is motivated by a morally irrelevant feature. But this would be to write off our very strong intuitions as irrational. It would be a puzzle why people who are mostly rational become irrational in these cases.

It must be conceded that some philosophers do disparage some of our intuitions as irrational, such as Peter Singer.^19^ But nevertheless, typically such philosophers try to reconcile their theories with our intuitions. For example, as Arras points out,^20^ Singer, in trying to defend his impartial theory in the face of our ordinary partiality for family and friends, says "There are impartial reasons for accepting some degree of partiality here."^21^ I will set aside the worries about whether his reasons are plausible, or whether they go far enough. The point is that, like the rest of us, he wants his theory to tally somewhat with our intuitions. Why think that a theory is a *moral* theory if it does not tally at all with our moral intuitions?

So I take it that moral theorists need to respect intuitions at least to an extent. I will outline one of the ways I operationalise this requirement. If I see that most people are strongly disposed to act in a certain way in certain conditions, because they are concerned about the harm that will result otherwise; and I see that they are disposed to criticise anyone who does not act in that way; and furthermore I see that they are even disposed to criticise those who fail to criticise those who do not act in that way; and finally I see they are disposed these ways even with full information and time to reflect—then I will take this as prima facie evidence that they are influenced by a valid moral principle. This does not mean I can't say they are mistaken—but then I would need to explain how they made the mistake.

But surely if identifiability is common to all rule of rescue situations, and yet is not a morally relevant condition, then our rule of rescue behaviour must be mistaken? Not if something else is *also* common to those situations, something that both drives our intuitions and justifies our behaviour. This is the possibility I will now consider.

## First morally relevant factor: Egalitarianism or Prioritarianism of life years

Nevertheless, identifiability could point us in the right direction. Rather than trying to defend the moral relevance of identifiability itself, Karen Jenni and George Loewenstein focus on a closely associated feature. They explain the "identifiable victim effect" in terms of the public preference for—or greater toleration of—wide rather than narrow distributions of risk.^22^ For example, studies have shown that people are more concerned about the risks of a vaccination program if only 10 per cent of the population are susceptible to adverse side effects, than if the whole population is susceptible, even if the number of people who will die will be around one thousand in either case.^23^ Jenni and Loewenstein hypothesise that the "identifiable victim effect" may be explained by this antagonism towards concentrated rather than shared distributions of risk. They report their own study which confirms this trend in public opinion, suggesting that "Subjects are significantly more concerned with saving lives when they represent a large portion of the reference group." For Jenni and Loewenstein, this suggests that

the major cause of the identifiable victim effect is the relative size of the reference group compared to the number of people at risk. Identified victims constitute their own reference group, 100% of whom will die if steps are not taken to save them.^24^

However, Jenni and Loewenstein have doubts about such intuitions, saying that "the normative arguments for a reference group effect are tenuous".^25^ One of their worries is that.

The reference group is often largely a matter of framing.^26^

They conclude that, as a general rule:

Given the arbitrariness of the reference group that applies to a specific risk, it seems inadvisable to recommend reference group size as an input into public policy.^27^

The issue here seems to be that we could frame our decision problem in terms of multiple different reference groups. Why should we assign someone to a small reference group with the implication that their risk is high rather than a big reference group with the implication that their risk is low? To choose either reference group might seem arbitrary.

However, reference groups need not be arbitrary. In ordinary healthcare decision-making we can estimate an individual's life expectancy based on everything we know about them, including demographics and comorbidities. Effectively each individual is assigned to their own notional reference group. We can do the same in the case at hand. Suppose we are considering giving 50,000 healthy people a vaccine against a fatal infection. If 10,000 of them have a genetic marker that we know is associated with a 10 per cent risk of dying, we can say that those 10,000 people have a 10 per cent risk of dying. On the other hand, if all we know is that 2 per cent of the population will die of the vaccine, and we have no other information as to who is at risk, then we will say that every individual has a 2 per cent chance of dying. In neither case is our assignation to a reference group arbitrary; in both cases, we appeal to all the risk-related information we have for each individual.

However, in addition, Jenni and Loewenstein also have a worry about a particular study which finds that the public distinguishes between

a situation where there is a group of 10 randomly distributed "vaccine sensitive" people who are at risk of death from a flu vaccine, but who cannot be identified beforehand, and a situation in which 10 random people will be killed by the same vaccine.^28^

Basically the public thinks that if the high-risk individuals *could* be identified in principle, they merit special concern, even if those individuals have not been identified at the time a decision is required. Jenni and Loewenstein do not think there is any good reason to prioritise such high-risk individuals over non-high-risk individuals who will nevertheless end up dying without the intervention.

This raises the question of whether we can defend distinguishing the two groups. I will set this question aside for a moment, coming back to it at the end of this section. But first: even if we cannot defend such a distinction, I believe Jenni and Loewenstein are conflating distinct cases here. The question at issue does not involve such in-principle-identifiable-but-unidentified individuals. Rather we are concerned with high-risk individuals who have been identified as such. We should start by asking whether we can vindicate the public's intuitive concern in these cases. Then, who knows, we may also be able to extend our analysis to high-risk individuals who have not yet been identified. But it will not be a fatal problem if we cannot. Even if we find that the public is mistaken in one case, that won't force us to conclude that they are mistaken in other cases.

But what morally relevant difference could there be between high risk groups and low risk groups? Of the well-known principles of distributive justice, the most obvious area to look is the egalitarian family of principles, including egalitarianism, prioritarianism and sufficientarianism. For example, focusing specifically on such principles within healthcare allocation, fair innings principles look like they could help. Fair innings principles favour patients who would die young without treatment, e.g. by up-weighting any life years we could add for those patients. Such principles appeal to intuitions that, for example, all else being equal, we have reason to give 5 years to a 40 year-old rather than 6 years to a 70 year-old, even though the older patient gets more benefit in terms of life years. I have argued that the concern motivating these principles is not based on the patient's age but on the patient's expected age at death: the point is that, without treatment, the patients in question will have had less life than others and they therefore have a greater need for additional life years.^29^ As Kappel & Sandoe put it, extending the lives of the old instead of the young "is like giving money to the rich instead of the poor".^30^ As such, fair innings principles are plausibly understood as motivated by an sufficientarianism of overall life expectancy, or of lifetime opportunity for welfare.

Perhaps some such principle favouring people expected to die young could also help explain some of our intuitions with respect to rescue cases. For example, consider a group of miners of mixed ages stuck down a mine, expected to die within days unless we can get them out. This will trigger rescue intuitions. Jenni and Loewenstein will explain this in terms of our intuitive aversion to the concentration of risk on the miners. I am suggesting that our intuitive aversion to this concentrated risk might be explicable in terms of the fact that without rescue, these miners can be expected to die younger on average than those not exposed to the risk, i.e. the rest of us. As such, the miners trigger something like a fair innings obligation; for example, perhaps when we calculate the cost-effectiveness of rescuing them we should weight the life years we could give them on the grounds that without a rescue they would have had fewer life years than the rest of us.^31^ We might then find that we are obliged to devote resources to rescuing the miners even if the same resources would produce more life years elsewhere. Thus a fair innings-style principle, favouring people expected to die young, can help vindicate our rescue intuitions with respect to the miners.

However, Bettina Schöne-Seifert has raised a counterexample against this sort of principle. If we prioritise people known to be high risk, then:

Rather than screening people susceptible for a potentially fatal disease and treat them early, effectively and at low cost one would wait until later—only to treat the very same patients at higher suffering, with higher risk and higher cost.^32^

Such a policy looks plainly irrational. However, there is a response to this. We should look for the people who are *actually* at risk, whether they are known to us or not. There are individuals whose physiology or environment or lifestyle puts them at risk, even if their high level of risk is not immediately obvious. Screening is justified because it enables us to identify those high risk individuals, who would otherwise be unjustly neglected despite their high risk.

This would also vindicate the public's concern for high-risk individuals who have not been identified, which we noted above.

So the lesson we can draw from all this is that identifiability was an imperfect proxy for what really matters, viz., underlying risk. But we can also explain why identifiability seemed to matter. In paradigm rule of rescue cases, the risk to which individuals are exposed is manifested in dramatic and vivid ways that are very motivating. This explains our sense of urgency in such cases. Nevertheless, the morally relevant features of such cases are also present in cases where the marks of risk are less obvious.

## Second morally relevant factor: that the negative outcome is sudden death

But unfortunately, fair innings-style principles do not appear able to explain all of our rule of rescue-related intuitions. For example, suppose Tony Bullimore or the Chilean miners had been 80 years old; the rescue services would not have made less effort to rescue them. And in healthcare, why do we spend money on treating older smokers with lung cancer rather than switching that cash to preventive measures which would produce more health benefit, and which would have stopped those individuals getting lung cancer in the first place, dramatically increasing their chance of a fair innings?^33^

My suggestion on this is inspired by the following finding regarding public opinion:

The study results suggest that [NICE's current end of life] policy may be insufficient in two ways. First, whilst it is concerned with patients’ remaining life expectancy, the supplementary advice does not distinguish between sudden and non-sudden disease progression. Findings from the pilot, coupled with an examination of the reasons given by respondents in the tick-box tasks, suggest that for many people the preference for prioritising the treatment of end-of-life patients is driven by concern about how much time the patients will have had to prepare for death.^34^

This is the common factor that would unite an 80 year-old Tony Bullimore or Chilean miner, as well as perhaps an older smoker with lung cancer. The suggestion is that all these individuals need time to prepare for their death. This is the reason we prioritise interventions that would help extend their lives, but not interventions that would extend the lives of patients who've had more notice of their death.^35^

To be clear what this implies, consider the choice between some 40 year-olds who have known since they were 20 that they were going to die in a month, and some 40 year-olds who have only just learned that they will die in a month of the same disease. If we have the choice between giving 10 extra weeks to the first group or 9 extra weeks to the second group, the principle I am suggesting would imply giving the 9 extra weeks to the second group, even though their health gain is smaller. I believe this is intuitively satisfying. Someone who has just been told they have a month to live is worse off than someone given more notice of their death, even if their death will have been at the same age and even if both will have had the same number of QALYs across their life. A fair innings-style principle favouring people expected to die young cannot account for this intuitive distinction, since both will die at the same age.

So, how to explain the intuitive distinction? The first group does have certain considerations on their side, i.e. fair innings-type considerations. But the second group has those considerations on their side and others as well; something to do with the suddenness of their imminent death. But why do we place weight on preventing deaths that come suddenly? Without an explanation, a principle in terms of the suddenness of someone's imminent death will look ad hoc.

In response, perhaps the most obvious explanation is the practical value. We recognise the value of people having time to sort out their affairs, make plans for the care of family members, say their goodbyes to friends and family, resolve longstanding disputes, and perhaps do that one important thing they’ve always wanted to do. So, the reason we favour one of the groups of 40 year-olds over the other is that in the favoured cases, the prospective rescuees have not had a chance to do the kinds of things most people get the chance to do as they approach death.

As an aside on this point, I should note an important feature of rule of rescue intuitions which I have taken for granted so far: they are much stronger in cases involving death than other cases. They seem to be much weaker in the case of most quality of life impairments. For example, consider a modified version [of the above case]. We must choose between some 50 year-olds who have known since they were 20 that they were going to get rheumatoid arthritis in a month, and some patients of the same age (50) who have only just learned that they will also get rheumatoid arthritis in a month. Intuitively, there is little reason to favour those who've only just learned about the imminent onset of the disease over those who have known about it for 30 years. If those who knew about it already were expected to get a bit more benefit, that could swing the decision their way (however, there might be an exception in the case of severely disabling impairments; I cover these below).

At first sight this distinction between imminent death and imminent quality of life impairments is puzzling. In assessing the benefits of a treatment we assess its effects on both life expectancy and quality of life. Why don't rule of rescue intuitions also treat both kinds of impairment equally?

My explanation is that there is relatively little benefit in being warned about an imminent quality of life impairment sooner rather than later; whereas in the case of one's imminent death, it makes a huge difference to be given some warning. For example, I contend there is a huge difference between the situation of a 30 year-old patient who learns he will die in a year and the situation of a 31 year-old patient who learns he will die in a week—even though they will both die at the same age. In contrast, there is much less difference between the situation of a 30 year-old patient who learns that their illness will cause lifelong disability in a year and a 31 year-old patient who learns that the same illness will give them the same disability immediately. Of course there is *some* difference between the two patients in the latter case; the patient who is given warning of their quality of life impairment can work out a bucket list of things which will be more difficult or less enjoyable after they get the impairment, and then get some of those things done. But this is also an advantage enjoyed by the patient given warning about their death over the patient given no warning of theirs. And the advantage enjoyed by the patient warned of their death is much greater than the advantage enjoyed by the patient warned of their quality of life impairment, since we cannot do any of the important things after we die, but we can do many of the important things after we get most quality of life impairments. In addition to this point, as argued above, the idea of sorting out one's affairs and making the right kind of exit from one's life is very important to most of us. This is a big advantage for the patient given warning of their death over the patient given no warning of theirs. There is no equivalent advantage for the patient given warning about their quality of life impairment over the patient given no warning of theirs. You do not need time to say goodbyes if you are going to be in a lot of pain in a month.

Returning to my main argument, I must now acknowledge that the practical considerations I have mentioned (sorting one's affairs; saying goodbyes) do not fully explain what is at stake. As it stands this is just a list of the things that patients facing imminent death would do if they were given more time. Other patients facing death, albeit with more notice, are also unable to do many things they would otherwise have done. Why are we so concerned with the things that patients given short notice of their death want to do?

I suggest that what motivates our concern is that we recognise a specific value in closing one's life story in the right way. We value life stories that follow certain patterns. Many of these are classic patterns which shape popular myths and stories. We are particularly averse to depriving people of the opportunity to follow some such widely valued pattern as they approach death.

In summary, the importance of these and other considerations can be explained by their narrative value. To elaborate, many writers have noted that in making sense of our lives and assigning them meaning, we resort to narrative conventions. Crossley provides a very helpful overview. She says:

the individual, at the level of tacit, phenomenological experience, is constantly projecting backwards and forwards in a manner that maintains a sense of coherence, unity, meaningfulness and identity.^36^

Summarising Sarbin, Crossley says:

human beings always seek to impose structure on the flow of experience. Such a narrative principle invokes a humanistic image of the self as a teller of stories, of heroes and villains, plots, and images of actors performing and engaging in dialogue with other actors.^37^

We think of our lives in terms of stories, perhaps because, as Carr says: "we are constantly explaining ourselves to others." What's more, "each of us must count himself among his own audience since in explaining ourselves to others we are often trying to convince ourselves as well."^38^

In line with this, one study shows how people in conditions of advanced cancer rely on narratives to come to terms with their lives and imminent death:

A meta-narrative of "saying goodbye in a good way" provided an overall structure for the participants as they attempted to create desired narratives negotiated in context of the individuals' sociocultural life and in the proximity of death.^39^

I suggest that sorting our affairs and saying our goodbyes are not just additional items to tick off from our lifelong To-Do list. Our imminent death should not just be a termination of the process of living but a trigger for the proper resolution of the story to that point. Specifically, if I am told I am about to die, two types of narrative may become important to me. The first is a story about me coming to terms with unresolved issues from my life to date. The second is a story about me coming to terms with my own imminent death.

On the first of these, namely the retrospective story, one thing I expect to do as I approach my death will be to think through my life. I will think of things that went well and things that didn’t. I may learn from this. One thing I may learn is to tell the story of an event differently. This raises the question of what it means to tell a story. Ricoeur says:

composing a story involves drawing together a series of events in order that they make sense in relation to one another.^40^

But how do we ensure that events "make sense in relation to one another"? I take this to mean that each event in a story explains or is explained by another event, either causally or evaluatively. We say that A happened because of B, or that A was important because of B, or that A throws light on B as an analogy. I am particularly interested in the evaluative judgments that may be elicited when we review our life as a story. A story could highlight that on balance, a certain choice was a mistake, or perhaps that I should not blame myself for what happened because I couldn't have known it would happen. Or, reviewing the big picture, I see that although event A was bad, it was worthwhile because of consequence B. These represent improved understandings. They may only be possible when we are close to death, when we no longer count ourselves as actors in the events in question or their aftermath.

But of course, if I am told I have a terminal disease, I won't just be interested in stories from the past, but also in the stories I'd told myself about my future, stories I can no longer live out. As Crossley says, traumatic experiences.

have the capacity to painfully highlight the ‘normal’ state of narrative coherence which is routinely taken-for-granted and thus remains ‘unseen’ within the active experiencing of everyday existence.^41^

However, now that I can no longer tell the same stories about my future, one way I can cope with my imminent death may be to find new stories I can tell about myself. Narrative can be used to.

restore a sense of order and connection, and thus to re-establish a semblance of meaning in the life of the individual.^42^

There is evidence for some of this in the behaviour of patients:

Research into the experience of chronic and serious illness illustrates the way in which our routine, ‘lived’ sense of time and identity is one of implicit connection and coherence. This sense is severely disrupted in the face of trauma and it is in such contexts that stories become important as a way of rebuilding a sense of connection and coherence. As the recent proliferation of autobiographies (especially in relation to diseases such as cancer and HIV/AIDS) and self-help groups suggests, for people suffering the trauma of illness, storytelling takes on a ‘renewed urgency’.^43^

So when we face death, we need time to review our life stories, relating to both the past and the future. Moreover, I suggest the very act of reviewing one's life stories is itself part of the overall story. It is a way of accepting one's death by closing the book on one's life. We achieve our full potential as human beings by realising what matters and setting aside what doesn't (such as unimportant disputes). Imminent death can prompt us to achieve such insights. Perhaps this achievement can (slightly) mitigate the badness of death.

In pursuing such insights, we seem to aim for a conventional narrative structure for our lives. Consider Christopher Booker's thesis that our favourite stories all follow seven basic plots.^44^ Most of these plots involve a hero who, through the events of the story, grows as a person. For example, by the end of a "Rags To Riches" story, the hero hasn’t just achieved material success, but also insight or a better character. In a romantic comedy, the protagonists don't just find a love partner but also learn lessons about what qualities they ought to value in people.

Now, at least in Western culture, this kind of personal growth is seen as an important element of our personal stories. Some people might even say it is what they are here for. However, most of us won't have achieved our full potential for growth by the time we face death. So, many people who know they are about to die will take this as a last opportunity to achieve some of this unrealised potential by getting a balanced perspective on their lives and on what matters. If some aspect of my life was bad but I have never fully faced that fact, I will get to face it, hopefully with some equanimity given the circumstances. If on the other hand there was something good about my life that I should have appreciated more, I will get one last chance to appreciate it. Alternatively, I may realise that a past event I've been angry about doesn't matter after all. And if my death will mean I never achieve success, whatever that meant—at least I can go to my death bed having fully confronted this important fact (whether I accept it or not). So, as in one of Booker's plots, at least by the end of the story, I can hope to have grown as a person.

And what of the second type of narrative, i.e. the story of one's life now, as one tries to comes to terms with one's own imminent death? One way of doing this may again involve making sense of it in terms of a story with a familiar structure. Consider Elisabeth Kübler-Ross's five-stage model of grief.^45^ The model says that we pass through five key stages of grief when we are facing our own death or that of a loved one: denial, anger, bargaining, depression, and acceptance. The interesting thing about this theory is how widely it has been accepted despite having been rejected by many of those who have assessed it carefully. In 1981, Dennis Klass was able to say that "Elisabeth Kübler-Ross’ five stage theory of grief is better known than all other thanatological writings combined."^46^ In 1994, Coolican et al. asked 650 nursing schools what models of death and dying they taught and 75% taught Kübler-Ross's model.^47^ In 2017, Stroebe et al. asserted that "It has remained hugely influential among researchers as well as practitioners."^48^ In 2020, Kübler-Ross’ book still ranked 107 out of around 1200 books in Amazon's Death & Bereavement section, 50 years after publication. Google returns 14 m results for a search of sites mentioning five stages of grief.^49^

So the five-stage model has widespread appeal. However, various studies have discredited the idea that there is a fixed sequence of stages of grief that we go through, or should go through. Among the many critics are Stroebe et al (2017), Corr (2019), Wortman (1989).^50^ All three complain about a lack of empirical evidence for the model. Stroebe et al. say the model does not help identify those at risk of complications in the grieving process. Both Stroebe et al. and Wortman object that different people grieve in different ways, and so the widespread expectation of these stages could prompt unhelpful interventions from others. Corr argues that even if people commonly experience these five ways of grieving, they do not do so in sequence but jump around between these responses, "sometimes simultaneously, sometimes repeatedly, sometimes with long intervals in between."^51^ According to Corr, Kübler-Ross herself accepted this fluidity and jumping around from one stage to another.

In light of these rejections of a fixed succession of universally experienced stages, including from the originator of the model, why is the simplistic version of the model so popular? I argue it is a manifestation of our need for certain narrative structures in coming to terms with death, and maybe even in assigning death itself a certain [qualified] positive value. In particular, the popularity of the five stages is a marker of our aspiration for life to be a progression. We might have stumbled in darkness for all of our lives so far, but at least at the point of death we want to go from darkness to light, and to turn from useless rejection of the inevitable to acceptance. In crystallising this aspiration, the five stages have something of Joseph Campbell's archetypal narrative structure.^52^ We are fighting through the initial trials, but we hope to gain insight and peace at the end. Suffering leads to wisdom.

Of course, that still leaves the worry that the five stages are something of a myth. For example, there are patients for whom they may represent an aspiration that will not be realised, since those patients go through a different sequence. No doubt there are other patients for whom the five stages are not even an aspiration, such as patients from non-Western cultures where death might be approached differently. But my point isn't that healthcare allocators should support the five stages specifically, but rather that the death of a patient should be respected in certain ways, not just afterwards but beforehand. We respect different people's funeral rites; we should equally respect different people's way of approaching death beforehand. And that partly involves giving people the time to approach death in whatever ways help them come to terms with it. The enduring popularity of the five stages is an expression of something more general, which is the human desire to approach death by doing things and experiencing things that constitute an adequate response to the seriousness of the event we are approaching. And for most patients, this isn't something that will take a day or two; it could take weeks or months.

It should be noted that what's important here may not be captured by a QALY calculation, or other framework based on maximising patient utility. Most people wouldn't be prepared to sacrifice life years for the sake of ensuring that their own funeral is a good one. Nevertheless, the rest of us should give the recently dead the right kind of send-off. Similarly with our rites as we approach the end of life. Perhaps none of us would sacrifice life years for the sake of an opportunity to enact those rites, but nevertheless it is fitting that we give that opportunity to people approaching their death.

In summary, narrative considerations can help explain both the practical value of having time to prepare for death as well as the cognitive or affective value of coming to terms with our life and our imminent death. Our aversion to letting people die suddenly is an aversion to depriving people of the opportunity to respond to their imminent death in the right way for them: whether it is sorting their affairs, saying goodbyes to family and friends, reviewing their life, or coming to terms with death itself.

## Two objections

I now consider two objections to this account. First, don't we also exhibit rescue behaviour towards animals? For example, consider the 1988 case of three California Grey whales stuck under the Arctic ice sheet, rescued after a massive operation involving multiple organisations and Soviet icebreakers.^53^ Given the similarity of our behaviour in human rescue cases and animal rescue cases, one might expect a common explanation in terms of the morally relevant considerations that drive our intuitions in these cases. But in the whales case, the rescuers won't have thought the whales should get an opportunity to go through Kübler-Ross's five stages, or an opportunity to put their house in order. So my explanation lacks the expected broad applicability.

To summarise the line I take in my response, I suggest that depending on circumstances it could have been morally acceptable to have let the whales die. In fact, some involved in the 1988 case "argued it was kinder to shoot them and put them out of their misery."^54^ This would never be proposed in the case of humans in a similar situation. This suggests that intuitively, we are not obliged to do as much for an animal in trouble as we would for a human in the same circumstances. For this reason, I do not think the same principles are at work in animal rescue cases and human rescue cases.

Nevertheless I must admit that the rescuers in this case felt like they had to rescue the whales (at least the central campaigners; perhaps some of the other parties had mixed motives). Given that I appeal to intuitions to support my account, this seems to pose a difficulty for me. Weren't some of the rescuers exhibiting "rule of rescue" intuitions?

To respond, I will start with a couple of bits of background. First, I need to clarify what I take a rule of rescue intuition to be. My working assumption throughout has been that rule of rescue considerations are moral considerations. I have been trying to explain why we may sometimes have an ethical reason to perform an expensive rescue even though the same resources would produce more benefit elsewhere. So a rule of rescue intuition is an intuition about what ethical reasons we have.

As further background, I now need to say something about the nature of ethical reasons, and relatedly how we can test for the presence of an ethical intuition. I won't attempt a complete characterisation of ethical reasons, but merely highlight an important feature for my purposes. To focus on the case in hand, when I discuss whether we can have ethical reasons to perform an expensive rescue, I am interested in reasons that anyone rational could recognise and respect (if I didn’t think such widely recognisable reasons existed, I would be less interested in writing about ethics). Well, given that ethical reasons according to my conception are widely recognisable, how could we determine whether or not someone's endorsement of a certain course of action is based on an ethical intuition? I suggest two tests of such an assessor's attitudes:

Test 1. Universal obligation to act: The assessor is disposed to criticise others in similar circumstances who do not do as the assessor would, and/or disposed to persuade others to do what the assessor would do.

Test 2. Universal recognisability: The assessor expects others to recognise the same ethical reasons, even as observers. This means the assessor is disposed to persuade/criticise anyone else who fails to persuade/criticise others in similar circumstances who do not do the same as the assessor would.^55^

According to my conception of ethical reasons, satisfaction of these two conditions is a necessary though not sufficient condition for someone's reasons to be ethical.

Now, returning to the main line of argument, my response to the whale case is that we often do things that are merely permissible and not obligatory. The question I address in this paper isn't whether we ever perform expensive rescues, but whether we ever have an *ethical reason* to do so. No doubt those who perform expensive rescues on animals generally feel quite strongly that they must help those animals. But this does not mean their attitudes are moral intuitions. As an analogy, suppose I come across a vet about to euthanise a lame fox in the Scottish countryside, on the grounds that there is no-one in the area who is willing or able to look after it and it will suffer if it is left alive. The euthanasia will not cause any suffering, but I look into that fox's eyes and feel sorry for it, and therefore object. The vet proves intractable. Then I might strongly believe that I must rescue that fox and look after it. But a strong feeling about what must be done is not necessarily a moral intuition. Even if I feel I must rescue the fox, I might not be disposed to persuade/criticise anyone else who is not disposed to rescue such foxes (eg the vet). And I may not be disposed to persuade/criticise anyone else who fails to persuade/criticise others who fail to rescue such foxes. So it would be perfectly consistent to feel I must help the fox without believing the vet would have been wrong to euthanise it. Thus my strong feeling that I must help the fox needn't be based on a moral judgment.

I believe we often have such nuanced attitudes in real world animal rescue cases. We might urgently want a stuck animal to be rescued, without thinking that a rescue is ethically obligatory. It seems that some of the rescuers in the whale case felt this way, initially suggesting the whales should be euthanised, but then helping with the rescue. Furthermore, if it was found that that a rescue would be extremely costly, such that the resources could produce more benefit elsewhere, I suggest few observers would have criticised the group if the group had decided to humanely kill the whales (not even observers who would have attempted a rescue themselves, given the opportunity).

So a disposition to rescue an animal does not necessarily derive from any intuition that it would be morally wrong not to rescue the animal.

And in fact, there are many conservationists who go further; they think it is wrong to rescue wild animals in trouble. For example:

Back in the 1990s, a strong windstorm blew bald eaglets out of their nest in Yellowstone. Park officials said that because it was a natural event, they were left on the ground to cope with whatever fate would come.^56^

This is not an isolated incident; in fact, this policy is supported by law:

a Canadian tourist captured a wild bison calf with his bare hands and loaded it into his SUV. The man, Shamash Kassam, said he found the animal alone and shivering along the roadside in Yellowstone’s wildlife-rich Lamar Valley. Once he turned it over to rangers, park biologists made several attempts to reunite the youngster with its herd but when the calf was rejected, they euthanized it. Kassam was fined $110 for violating park regulations, which strictly forbid contact with wildlife.^57^

Thus it does not look like there is any need for me to extend my account to the whales case, because even though we might want the whales to be rescued, it is not clear that we are *morally obliged* to rescue them. There are many who would think it was permissible not to rescue them in cases where the costs were very high (these being the cases analogous to the human cases I am considering). In addition, there are some very knowledgeable people, i.e. conservationists, who would say it was wrong to rescue them.

The human case is very different. Suppose that in Chile the prospective mine rescuers had decided that for cost reasons, they were going to humanely kill the Chilean miners instead of trying to rescue them. Then, there would have been outrage in Chile and around the world, probably combined with direct action to prevent the plan being implemented. Thus there is a stark contrast between our attitude to animal rescue cases and our attitude to human rescue cases. There is a disposition to rescue in both cases, but there is little disposition to criticise non-rescuers in the animals case. I suggest this reflects the very different moral status of the two sets of cases.

Having said that, I do not deny that it is *sometimes* obligatory to rescue an animal in trouble. But I contend that such cases will be explicable by other factors that are not relevant to the high-cost rescue cases I am trying to explain. For example:

**Disanalogy 1.** Cases where rescuing an animal would be the most beneficial use of resources. Such rescues are not of the puzzling type I am considering, where the resources deployed in the rescue could produce greater benefit elsewhere. Rather, once we assume that animals can gain utility, these "cost-effective" rescues are justifiable under one of the most popular evaluative frameworks for policy-making, a utilitarian-style framework.

It is therefore no objection to my account to point out that we often expend great energy rescuing animals. A decisive objection would need to pick out a certain animal rescue which we would all take to be morally obligatory, and pick out a compelling measure of benefit or utility, and then show that the rescue would fail to maximise benefit on that measure (and that the rescue does not involve any of the other confounding factors mentioned below).

**Disanalogy 2.** Rescues that prevent extreme suffering. Any prevention of suffering is a benefit, whether human or animal suffering. Rescuing an animal may be the most effective way of preventing suffering. As above, if a rescue would be the most beneficial use of resources, then the question I am addressing does not arise.

**Disanalogy 3.** Rescuing pets and other human-owned animals. I argued above that it might be permissible not to rescue wild animals. However, I must now admit that we sometimes devote significant resources to rescuing human-owned animals. My response is that we have plenty of reasons to do this apart from the kind of obligations I've outlined. I will offer two such confounding factors. First, given that human-owned animals are captured or brought into existence by humans to serve human purposes, it can be argued that the community that benefits from the animals jointly incurs a obligation to go to great lengths to ensure their welfare, somewhat analogous to the obligations that parents incur towards the children they bring into existence. And second, if the rescuers are public servants, as is commonly the case, their reason for rescuing the animal is not just that it benefits the animal, but because taxpayers pay for those public servants to provide this service to animal owners, as well as the wider public who benefit from the existence of the animals. In fact, even if a rescuer is not a paid public servant, their efforts can be seen as part of our duty to show concern for each other's welfare—so, even if an animal rescuer's extreme exertions could have produced more benefit elsewhere when judged solely against the amount of benefit to the animal, the rescue may become cost-effective when we also consider the human beneficiaries.

To illustrate the importance of human beneficiaries in such cases, I will try to devise a hypothetical case involving an expensive rescue, of a not-entirely-wild animal, which is not human-owned, and which is not suffering. Imagine a feral cat whose remote ancestors were escaped domestic cats. This cat has fallen off a cliff-face half-way up a mountain. It is now lying unconscious on a ledge. Unless we mount a £500 k rescue, it will remain unconscious until it freezes to death overnight. I suggest no-one would criticise the local authorities if they do not mount the rescue. In contrast, if someone owned the cat, then perhaps there would be more criticism for a failure to rescue it—if so, this must be explained by duties arising from the human ownership of the cat, eg duties to the owner, and not only duties to the cat (however, please note: my arguments in the rest of this paper do not *depend* on this particular rescue being morally obligatory). Finally, for completeness, consider the case of a shepherd lying unconscious after falling to the same ledge. Even if a rescue would not be cost-effective by normal standards, I suggest there would be strong, widespread criticism if local authorities failed to mount a rescue—much more criticism than even in the case of the owned cat. This human case is the kind of case I am trying to explain.

I conclude that there are specific, morally relevant considerations to explain why we should rescue human-owned animals, making it unnecessary for me to try and extend my account to them.

Disanalogy 4. Rescuing animals who have been harmed by something man-made, eg a seagull caught in fishing line or a toad caught in discarded plastic tubing. If certain humans are the cause of the problem, they have a responsibility to sort it out; and if the culprits do not fulfil their responsibility, perhaps that responsibility passes to other humans.^58^ The same consideration applies to animals that have been exploited, eg for entertainment, and are now suffering or neglected.

Disanalogy 5. Rescuing members of rare species. We have more reason to rescue an animal if its species is at risk of extinction. This is reflected in wildlife policy: "species considered common are less likely to be candidates for intervention".^59^

So I contend that in many cases where we feel that we must rescue an animal at great expense, we do not have any ethical reason to do so. And in cases where we *do* have an ethical reason to perform an expensive rescue on an animal, there is a special factor in play which is not seen in the human cases I am trying to explain. So there is no need for me to extend my account to animal cases, and no prospect of deploying the morally relevant factors seen in animal rescue cases as a basis for explaining our obligations in all cases where we have an ethical reason to perform an expensive rescue of humans.

For the second objection, it may be objected that not everyone will want notice of their death. Some would rather die of a heart attack in their sleep, even if they haven't made any preparations for death. However, I would respond that most people would want to know they are dying, presumably for the reasons I have given.^60^

Furthermore, it should be noted that the question at issue isn't whether to give bad news to patients who don't know, but whether to offer more time to patients who do know. Even if some patients who've had the bad news would have been better off not knowing, it is not inconsistent to claim that, now they know, a bit of time to prepare is very important for them.

Finally, even if it is true that most people want a quick death, again, what's important may not be captured by a QALY calculation. It's about fellow citizens expressing respect and compassion for those who are about to die. This means giving extra time to patients who want it. This is not to say that considerations of cost-effectiveness don't come into it—if only 1% of a population want extra time and 99% don't, that weakens the case for policy-makers to implement special measures. My suggestion is merely that we shouldn't *only* assess such measures based on their value to patients themselves.

So, for someone who has not been given much notice of their death, all else being equal we have greater reason to give that patient an extra month than other patients. To an extent, the quality of the patient's whole life may be caught up in that month, in that some of the stories of the patient's life can end up better or worse according to whether the patient is given a chance to end them properly. The narrative-closing rituals that people enact as they approach death are as important as the funeral rites we enact after their death. We should be as averse to denying patients this opportunity as to putting a patient's body into the waste disposal or preventing relatives from holding a funeral. That does not mean we should never do it; the 2020 Covid-19 outbreak demonstrated that even family goodbyes and funerals are not entirely sacrosanct, with families prevented from meeting Covid-19 patients in their last days and attendance at many funerals restricted to six. But the circumstances need to be extreme to justify such measures. It is notable that when such lockdown measures were introduced, they were then eased very early, at least in the UK.^61^

## Conclusion: implications for health policy

I have explained our rule of rescue intuitions in terms of a plurality of principles, which have various implications for healthcare. Where a patient is expected to die young, fair innings principles imply that we should relax our cost-effectiveness criteria and prioritise that patient. This justifies much rule of rescue-type behaviour. In addition, I have argued that we should ensure that people have notice of their death so they have time to prepare. This gives us reason to prioritise life-extending treatment for those who are not expected to live long after the diagnosis of the condition that would kill them if not treated.

In many cases, these two considerations will be mutually supportive. But in many cases, rescue-type behaviour may be solely motivated by my principle that people should be given reasonable notice of their death. How does my principle compare with existing policies? In the UK, NICE's end of life premium looks like it might be motivated by somewhat related considerations. I will evaluate the end of life premium as if it is an attempt to capture rule of rescue considerations (ignoring for the sake of argument the possibility that it was not motivated by such considerations).

NICE's end of life premium gives priority to patients satisfying the following conditions:

1. The treatment is indicated for patients with a short remaining life expectancy, normally less than 24 months and;
2. There is sufficient evidence to indicate that the treatment offers an extension to life, normally of at least an additional 3 months, compared to current NHS treatment, and;
3. The treatment is licensed or otherwise indicated, for small patient populations^62^

When the above conditions are met, NICE's Appraisal Committee is advised to consider:

- The impact of giving greater weight to QALYs achieved in the later stages of terminal diseases, using the assumption that the extended survival period is experienced at the full quality of life anticipated for a healthy individual of the same age, and;

- The magnitude of the additional weight that would need to be assigned to the QALY benefits in this patient group for the cost-effectiveness of the technology to fall within the current threshold range.

Thus policy-makers are given quite wide discretion in how they respond to the above conditions. Nevertheless, the "direction of travel" is clear. Firstly, within limits they can to give greater weight to QALYs gained in these circumstances (in that they can act as if more QALYs are gained than actually are). Secondly, the QALY calculation can be based on an assumption full quality of life, so if patients satisfying the above condition have poor quality of life, that will not negatively impact the deemed cost-effectiveness of their treatments.

This policy has had an impact:

NICE has applied EoL flexibilities in 25 TAs since the guidance was introduced. Of those, 18 have resulted in NICE recommending use … what really matters is the cost-effectiveness threshold used when [Appraisal Committees] consider these treatments. The magic number, based on an average across all positive recommendations, seems to be around £49,000 per QALY.^63^

This compares with the normal threshold of £20,000—£30,000. Thus the effect of this policy has been to relax the cost-effectiveness threshold for life-extending drugs.

How does this policy look from the perspective of the principles I have defended above? I will focus on the first two conditions in NICE's policy, and with a view to clarifying the implications of my account, I will compare NICE's policy with my principle stipulating priority for life-extending treatments for patients who lack reasonable notice of their death.

In making my principle more precise the first question would be: how should the life expectancy terms be specified? NICE defines its timings in terms of how long the patient is expected to live as at the time of the treatment decision. As seen from the perspective of rule of rescue obligations, this must be seen as a mistake. If I am right that part of the point of a policy like this is to ensure that people have reasonable notice of their death and time to sort out their affairs, someone who has known for many years that they can expect to die this year should not be treated the same as someone who has only just learnt. To preclude such cases, the timings would need to be specified in terms of time from diagnosis. The question is, how long from diagnosis can the patient expect to live without treatment and how long they can expect to live with treatment? On the basis of medical advice, or commonly known information about ageing, an elderly patient should have realised for some time before their death that their death was imminent.

The next question is, how long does someone need to sort out their affairs? Of course the longer the better, and it will be difficult to generalise, but I would hypothesise that after a few months, a law of diminishing returns kicks in. Without wishing to sound harsh, after a year, even someone with a complicated life should have been able to sort their affairs. So, NICE's stipulation that anyone with less than two years to live qualifies for special treatment would seem to be slightly on the generous side, from the perspective of rule of rescue obligations. But of course, there are no sharp lines on this question, and anyway empirical research would be required to answer it properly.

Having made this decision there will be another, closely related decision, which is the question of how many extra years produced by treatment qualify for the premium. The same reasoning implies the same limit: whatever time is needed to sort out one's affairs, that should be both the maximum remaining life expectancy without treatment to qualify for the premium, and also the maximum life expectancy *with* treatment to qualify for the premium. There should be no weighting for life expectancy improvements above the period deemed necessary to sort out one's affairs. If a treatment produces more years, of course they count as a benefit, but they are not specifically needed for someone to sort out their affairs, and therefore should not qualify for a premium which is specifically motivated by that need.

So the value of an extra month or year depends on the context: an extra month is worth a lot for someone who has just been diagnosed and who is only expected to live a month without treatment. But it is worth less for someone who is expected to live two years without treatment.

Now consider NICE's stipulation that to qualify for the premium, a treatment needs to produce at least an extra three months. But this misses out perhaps the most valuable period that a terminal patient could be given. If I have been given a week to live and a treatment promises to add another week on top, that is a huge difference. The requirement should only be that a treatment adds *something*.^64^ Also in relation to this point, I would suggest that the weightings stipulated in the policy should be graduated. The first month or two after diagnosis are extremely valuable, both for allowing the patient to come to terms with their death and for allowing them to sort out their affairs. Thereafter, returns diminish. I would propose that if a patient is expected to live less than, say, three more months, the weightings for additional life expectancy within that period should be high. Thereafter, the weightings should get lower.

However, perhaps imminent death is not the only thing that should trigger such measures; perhaps we should also prioritise any patient facing an imminent disabling state (such as a coma) that will prevent them approaching death in the right way. Suppose a patient faces the imminent loss of their mental capacities, or a coma that will last the rest of the patient's life, such that they won't be able to get any of the important things done that they would want to do before they die, such as communicating with family and friends.^65^ Then, on my account, we might have the same case for prioritising that patient as if they were facing death. So my suddenness consideration is gradable; it carries more or less weight according to the nature of the imminent condition. With non-disabling conditions it carries no weight, but with any completely disabling condition it carries a lot of weight.

In addition, my principle should also be understood as implying priority, not only for those clearly at risk, but also for those who are at non-obviously at risk, where such individuals can be picked up with screening.

Finally, in terms of preventive interventions for healthy patients, my principle implies that the priority should go to heart attack prevention rather than preventing slow growth cancers which get detected early, other things being equal.^66^ Heart attacks kill suddenly, whereas a patient who learns early about a slow growth cancer has notice of their death. Perhaps this implication favouring heart attack prevention is somewhat counterintuitive, but not unacceptably so. It is an acceptable cost for an account which has otherwise intuitively satisfying implications. Intuitions are not infallible. The account's intuitively satisfying implications in other cases give us reason to rethink our intuitions in this case and/or see them as mistaken.

In summary, if NICE's end of life premium is seen as an attempt to capture rule of rescue considerations, it falls short in respect of several important details. These include the stipulation of how short life expectancy must be to trigger priority, and the definition of the moment from which life expectancy is measured. The policy also omits certain patients who merit priority, such as patients facing lifelong comas. Perhaps the reason is that, although NICE correctly noticed that imminent death can trigger rule of rescue intuitions, NICE did not fully appreciate *why* imminent death triggers those intuitions when it does. I hope I have thrown light on that question.

## Footnotes

1 Burrell (1997).

2 Farnsworth (1997).

3 Triggle (2005).

4 Hadorn (1991).

5 Hadorn (1991).

6 Hadorn (1991).

7 Harris (1987) quoting BBC^1^ (1986).

8 It might be objected that it is more likely a patient will be helped by dialysis than a smoker will be helped by a stop smoking campaign. But any such difference of probabilities is accounted for in the QALY estimates, eg a 50% chance of two QALYs is counted as one QALY.

9 Identifiability needs defining, since it comes in more than one form. We can't insist that identifiability amounts to a prospective rescuer knowing who the prospective rescuee is, since in many paradigm rescue cases, the prospective rescuee is not known to the prospective rescuer in this sense. I will assume that a prospective rescuee is relevantly identifiable if someone else is able to give a non-tautologous description that picks them out, knowing that everyone satisfying that description is uniquely at risk of imminent death. An example of a non-tautologous description is one that specifies the person's location. So I am identifiable in this sense if I am stuck down a mine facing imminent death and you know that there is someone stuck down that mine facing imminent death, even if you don't know my name and can't see me. (To explain the "non-tautologous" bit: someone facing imminent death is not relevantly identifiable just because I know that there is someone facing imminent death who is facing imminent death).

10 Hadorn (1991). Other writers who take identifiability to be the condition that triggers rule of rescue behaviour include Cookson et al. (2008) and Hope (2004). Also McKie & Richardson (2003)—but they go on to make a normative case *against* identifiability playing this role.

11 McKie & Richardson (2003)

12 Orr & Wolff (2015).

13 Largent & Pearson (2012).

14 Schöne-Seifert (2009).

15 Sheehan (2007). Actually in a private communication, Sheehan clarifies that he only wants to "make space" for the role of identifiability in an agent-relative rescue obligation, without offering an all-out defence.

16 McKie & Richardson (2003).

17 IFRs tend to involve small patient populations for which there is less data available.

18 Admittedly, the data is often not robust, because of the small patient populations. But there is usually *some* data, and if there is enough data to draw some very approximate conclusions about cost-effectiveness, it would not seem impermissible to do so, albeit allowing for large margins of error.

19 Singer (2005).

20 Arras (2016).

21 Singer (2004).

22 Jenni and Loewenstein (1997). Thanks to Rob Lawlor for pointing me to this way of vindicating the concern with identifiability in terms of a concern with risk.

23 Ritov & Baron, 1990

24 Jenni & Loewenstein (1997).

25 Jenni & Loewenstein (1997).

26 Jenni & Loewenstein (1997).

27 Jenni & Loewenstein (1997).

28 Jenni & Loewenstein (1997).

29 Sinclair (2019).

30 Kappel & Sandoe (1992), p. 313. However they then make the common mistake of defending a principle expressed in terms of patient age rather than expected age at death.

31 I have not tried to explain our intuitive aversion to concentrated risk in Jenni and Loewenstein's vaccine case here. I believe that it can be explained by a similar egalitarianism of life years or lifetime opportunity for welfare. However, some of the fair innings principles that have been canvassed do a poor job in the vaccine case, since those principles only imply priority for patients who, without our intervention, will be worse off than others (e.g. see Williams, 1997). In Jenni and Loewenstein's case, without our intervention, no-one will be worse off than others; all die. The inequality at issue is inequality produced by a prospective intervention, rather than a background inequality. An egalitarian of lifetime opportunity for welfare should be as concerned about this outcome inequality as they would be about background inequality, but must express that concern differently. However it is not easy to formulate a good principle. One possibility is to say that when we are comparing two or more interventions with similar benefits, we should down-weight all the benefits of each intervention proportionate to the extent its benefits would be enjoyed unequally among those who need such benefits.

32 Schöne-Seifert (2009), p. 424.

33 For example, one study suggested we could get 10 QALYs from dialysis of kidney patients or 1197 QALYs from stop-smoking campaigns. See Harris (1987) quoting BBC^1^ (1986).

34 Shah et al (2014), p. 397.

35 In the following discussion, I set aside patients whose quality of life is worse than death, and who would therefore not want their lives extended.

36 Crossley (2000).

37 Crossley (2000) quoting Sarbin (1986).

38 Carr (1986) cited in Crossley, 2000

39 La Cour et al. (2009).

40 Crossley (2000) quoting Ricoeur (1991).

41 Crossley (2000).

42 Crossley (2000).

43 Crossley (2000) quoting Mathieson & Stam (1995).

44 Booker (2004).

45 Kübler-Ross (1969).

46 Klass (1982).

47 Coolican et al. (1994).

48 Stroebe et al (2017).

49 This is based on a search of: *grief "five stages"*, with the latter two words in quotes.

50 And Corr cites half a dozen others.

51 Corr (2019).

52 Eg see Campbell (1949).

53 Leonard (2012). I thank an anonymous reviewer for referring me to this case.

54 Leonard (2012).

55 These tests are inspired by the work of various philosophers and moral psychologists on the universalisability of moral judgments. As a source for my first test, perhaps Hare (1952, 1991) is best known for the suggestion that ethical sentences function similarly to universalisable imperatives. And once the first test is seen as appropriate, perhaps the second test on the universal recognisability of moral considerations will also seem appropriate: at least for a liberal, it would not seem acceptable to universalise an imperative unless those on the receiving end of it could in principle recognise its validity. As another source for the second test, consider Blair's (1995, 1997) work on the "moral/conventional" distinction. As Gill & Nichols (2008) summarises it: "Children judged hitting to be more seriously wrong than talking in class. Children typically said that hitting would be wrong even if the teacher had no rule against it [moral], but they were less likely to say that about talking in class [conventional]." It seems that children will judge hitting to be wrong even without authority figures telling them so. This suggests that anyone of a certain maturity could see that hitting is wrong, and this is partly what enables us to see the principle debarring hitting as a moral principle.

56 Wilkinson, (2016)

57 Wilkinson (2016).

58 Animal Ethics (2021).

59 Wilkinson (2016).

60 See Sullivan et al. (2001), Tuckett (2004), Hagerty et al (2005), Fallowfield et al. (1995), Higgs (1982).

61 HM Government (2020).

62 NICE (2009).

63 Barham (2016).

64 Of course if it only adds an hour, there will be very little benefit to weight, so even the weighted benefit will be minimal.

65 Thanks to Rob Lawlor for this point.

66 But of course, other things might not be equal. Having a slow cancer, and living with the knowledge you are about to die, might be a consideration that runs counter to the considerations I have outlined. Thanks to Rob Lawlor for this point.
